# Leiomyosarcoma of the Inferior Vena Cava - Radical Resection, Vascular Reconstruction and Challenges: A Case Report and Review of Relevant Literature

**DOI:** 10.4021/wjon471w

**Published:** 2013-05-06

**Authors:** Saptarshi Biswas, Arpit Amin, Suhaib Chaudry, Saju Joseph

**Affiliations:** a Department of General Surgery, Westchester University Medical Center, NY, USA; b Department of Surgical Oncology, St Vincents Medical Center, Bridgeport, CT, USA

**Keywords:** Inferior vena cava, Leiomyosarcoma, Radical resection, Vascular reconstruction

## Abstract

Leiomyosarcomas of the inferior Vena Cava (IVC) are rare soft tissue sarcomas accounting for only 0.5% of all soft tissue sarcomas in adults with fewer than 300 cases reported. Extraluminal tumor growth along the adventitia of the IVC seems to be the common presentation. Intraluminal tumor growth is rare. The origin of the tumor is divided into three levels in relation to the hepatic and renal veins. The presentations and surgical modalities vary accordingly. Retroperitoneal tumors are often not diagnosed until the disease is at an advanced stage with large tumor growth and involvement of surrounding structures. This is partly because of the nonspecific clinical presentation as well as absence of early symptoms. Most patients present with abdominal or flank pain. Symptoms vary according to the dimensions of the tumor, growth pattern and localization of the tumor. Radical en bloc resection of the affected venous segment remains the only therapeutic option associated with prolonged survival. The goals of surgical management of these tumors include the achievement of local tumor control, maintenance of caval flow, and the prevention of recurrence. The involvement of renal or hepatic veins determines the strategy for vascular reconstruction. Reconstruction of the IVC is not always required, because gradual occlusion of the IVC allows the development of venous collaterals. However, when pararenal leiomyosarcoma of the IVC is present, reconstruction of the IVC and the renal vein is necessary to prevent transient or permanent renal dysfunction. Recent study has shown that radical surgery combined with adjuvant multimodal therapy has improved the cumulative survival rate. We report a case of IVC leiomyosarcoma in a young healthy woman along with details of its diagnostic workup and discussion of the surgical options and reconstruction of caval continuity.

## Introduction

Primary vascular leiomyosarcoma is a rare tumor of mesenchymal origin and arises from the smooth muscle cells of the tunica media predominantly within the inferior vena cava [1, 2]. Extraluminal tumor growth along the adventitia of the IVC seems to be the common presentation [1, 3]. Intraluminal tumor growth is rarely found.

The origin of the tumor is described in relation to the hepatic and renal veins. The IVC is divided into three levels [1, 4-6]: a). Level 1 extends from the entry of the hepatic veins up to the right atrium; b). Level 2 comprises the area between the confluences of the renal and hepatic veins; c) Level 3 includes the area below the renal veins.

Retroperitoneal tumors are often not diagnosed until the disease is at an advanced stage with large tumor growth and involvement of surrounding structures. This is partly because of the nonspecific clinical presentation as well as absence of early symptoms. Most patients present with abdominal or flank pain [1, 4].

Radical en bloc resection of the affected venous segment remains the only therapeutic option associated with prolonged survival [1, 4, 5]. Ito et al in a recent study with leiomyosarcoma of the IVC concluded that radical surgery combined with adjuvant multimodal therapy had a 5-year cumulative survival rate of 62% [6].

The goals of surgical management of these tumors include the achievement of local tumor control, maintenance of caval flow, and the prevention of recurrence. The involvement of renal or hepatic veins determines the strategy for vascular reconstruction.

Reconstruction of the IVC is not always required, because gradual occlusion of the IVC allows the development of venous collaterals. However, when pararenal leiomyosarcoma of the IVC is present, reconstruction of the IVC and the renal vein is necessary to prevent transient or permanent renal dysfunction [1, 5, 7].

We report a case of leiomyosarcoma of the IVC with discussion of the surgical procedure and reconstruction of caval continuity.

## Case Report

A 47-year-old previously healthy female presented with abdominal pain and generalized discomfort over a span of 4 to 6 months. The patient had no exacerbating or alleviating factors. The respiratory, cardiovascular, and CNS system review was essentially normal.

The abdomen was grossly soft, with no rigidity or guarding. Upon auscultation, bowel sounds were audible in all four quadrants. On deep palpation, there was a mass in the right upper and right mid quadrant with overlying mild tenderness.

Her past medical history included nephrolithiasis and gastroesophageal reflux disease. Previous surgical history was significant for bilateral tubal ligation. Her family history was notable for bladder cancer in her mother. The patient denied taking medications or having any allergies. She was married, lived with her 3 children, and denied smoking, alcohol, and illicit drug use.

Due to persistent discomfort, she was evaluated by gastroenterology by an upper endoscopy, ultrasound of the abdomen, and colonoscopy which were all unremarkable. The preliminary labs included a CBC and a Basic Metabolic Panel which were essentially within normal limits.

### Radiology work up

**Figure 1:**
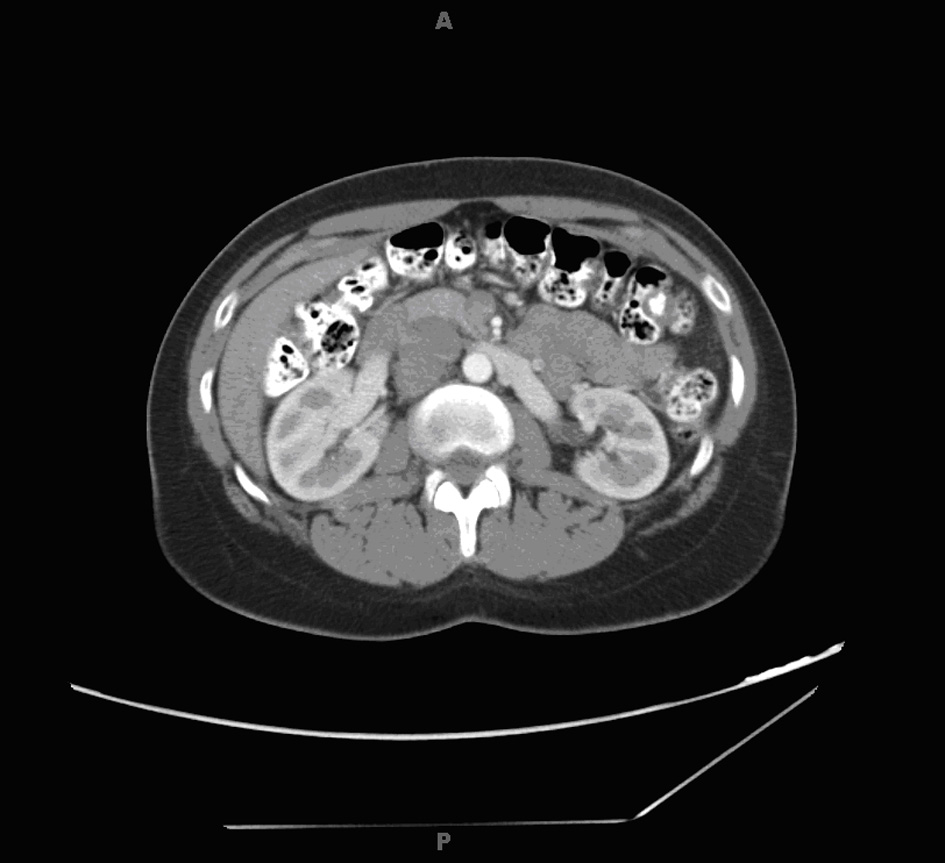
Abdominal computed tomography scan showing right paracaval mass.

Radiology work up included a CT scan of the abdomen and pelvis which revealed a 4.6 × 3.6 × 3.9 cm right paracaval mass extending to the right renal vein and artery alongside effacement of the inferior vena cava (Fig. 1). In addition, an enlarged and lobulated uterus was visualized in the pelvis.

A CT guided biopsy was performed of the retroperitoneal/pericaval abdominal mass, and multiple core samples were obtained. Initial light microscopy evaluation of the sample demonstrated evidence of atypical spindle cells however, the precise type of cancer could not be determined on this exam. The core samples were sent to pathology for additional evaluation.

A PET imaging scan was performed from the skull base to the mid-thigh with concurrently acquired low-dose CT for attenuation correction and anatomical localization. The scan showed a heterogeneous area of increased radiotracer activity in the upper right retroperitoneum in the region of the IVC. A large focus of increased radiotracer activity was noted along the right side of the uterus, more intense than usual for a benign fibroid. There was otherwise normal, physiological distribution of radiotracer activity throughout the visualized portion of the body. No other focal areas of abnormally increased activity were identified.

### Operative procedure

After obtaining sufficient evidence that there were no pulmonary or hepatic metastases, the patient was cleared as a candidate for surgery. The small intestine and large colon were mobilized and lifted out of the way. The large mass was identified in the retroperitoneal space. The liver was mobilized, and dense attachments to the gallbladder were taken down.

**Figure 2:**
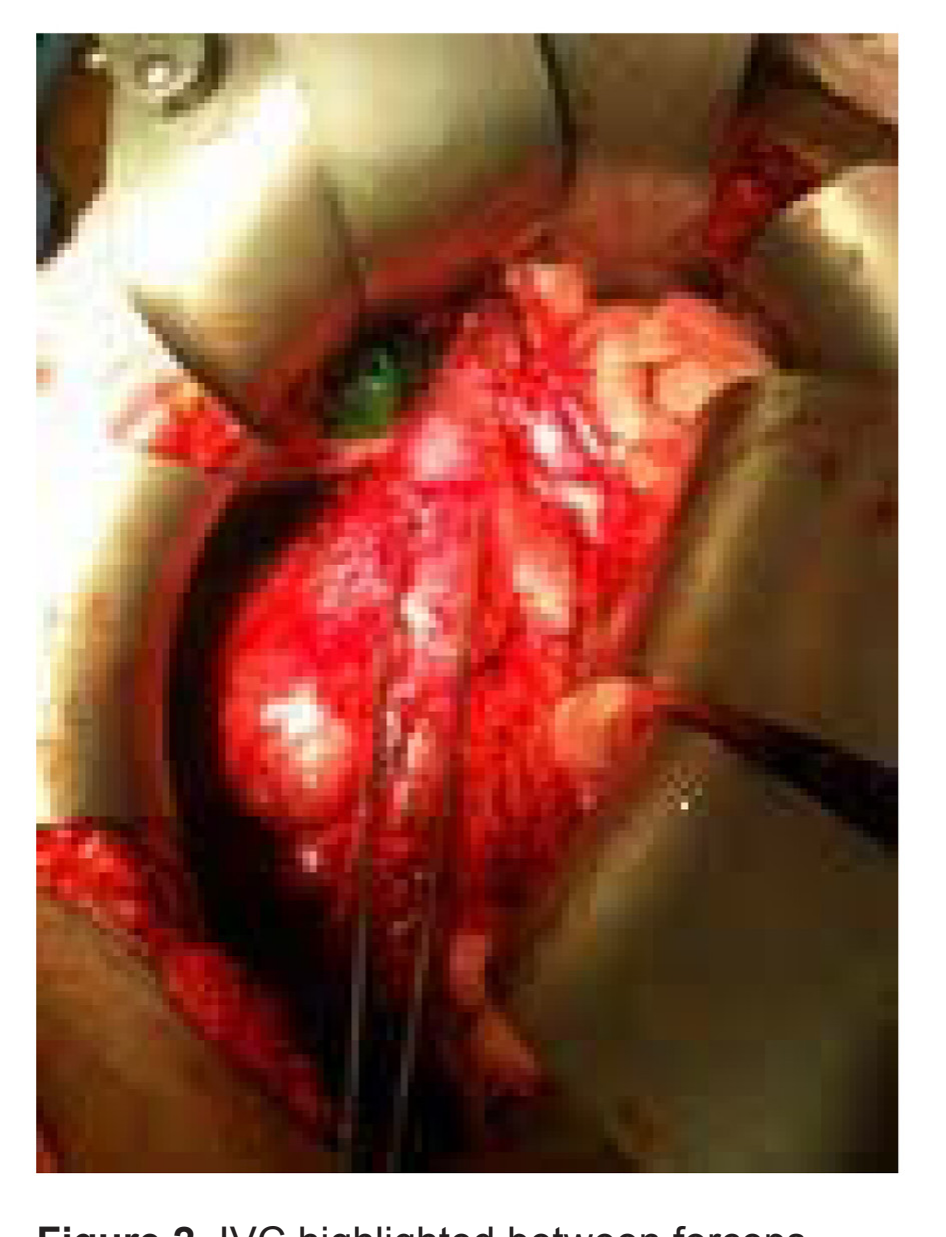
IVC highlighted between forceps.

The inferior vena cava was completely mobilized and encircled near the iliac bifurcation giving us distal control of the inferior vena cava (Fig. 2). The aortic caval space was opened and para-aortic lymph nodes were removed all the way up to the level of the SMA. A vessel loop was used on the inferior vena cava above the level of the renal vessels, giving us proximal control. The kidney was mobilized off the retroperitoneum. The tumor was mobilized off the posterior spine. The right renal vein was visualized which was posterior to and encased in the tumor. After ligating the renal artery, the tumor was completely mobilized off the inferior vena cava using sharp and blunt dissection. The left renal vein was dissected out and preserved as was the suprarenal as well as the infrarenal vena cava just above the level of the bifurcation.

**Figure 3:**
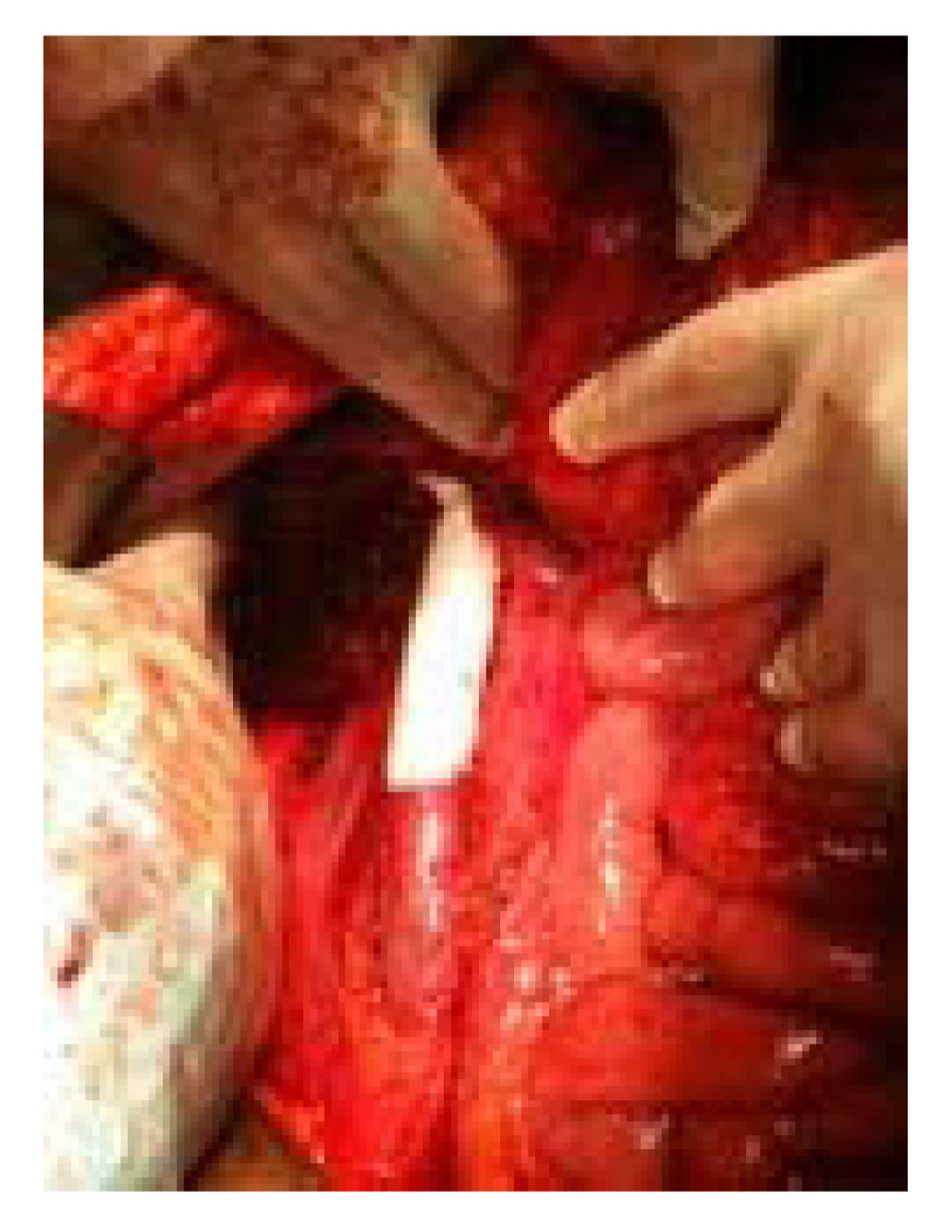
IVC reconstruction with Gore -Tex vascular graft.

The tumor, as well as the right kidney was resected off the IVC en bloc using a bevelling technique to preserve the orifice of the left renal vein within the confluence of the suprarenal cava and then transecting the infrarenal vena cava just above the bifurcation. With the tumor removed, a 20 mm Gore-Tex graft was then doubled approximately and an end-to-end anastomosis was completed using running 4-0 Prolene (Fig. 3). This anastomosis, which included the renal vein on the left and the suprarenal cava, was tested, and flow was restored from the renal vein into the vena cava. After appropriate flushing maneuvers were performed, flow was restored and excellent flow was noted. Frozen section margins of the specimen were negative. Due to the concern for a secondary malignancy, a total abdominal hysterectomy was performed.

### Pathology

The closest margin was confirmed to be less than 1 mm and consisted of multiple mitotic figures per high-power field. The patient was subsequently classified as T1b N0 M0 AJCC stage IIA.

**Figure 4:**
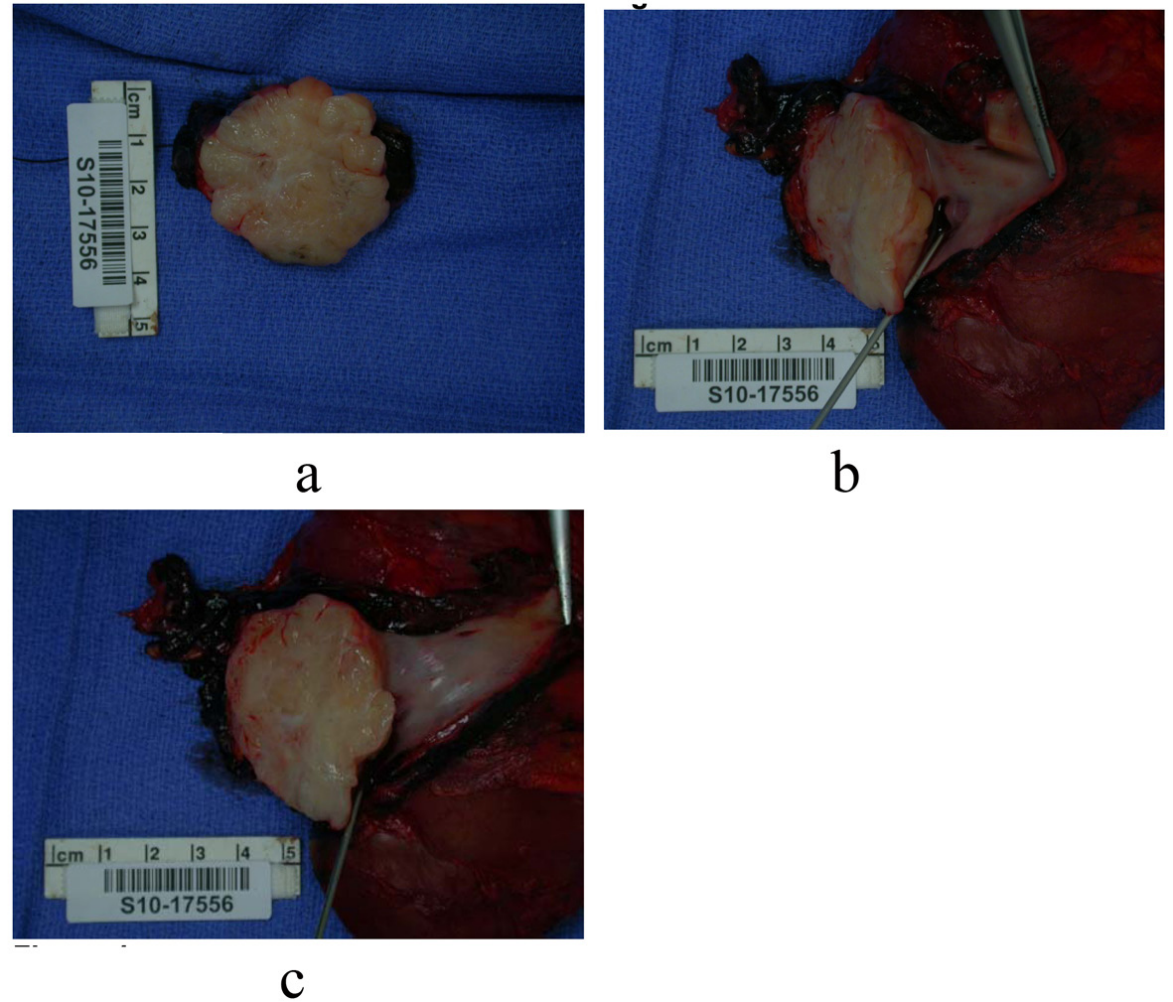
(a - c): Intraoperative photographs of the gross specimen.

A radically resected retroperitoneal/Inferior vena caval specimen was submitted to pathology for evaluation. The soft tissue was fresh, 251 grams in weight, and 11.5 × 6.5 × 4.0 cm in dimensions. On sectioning the IVC, the cut surface showed a 4.5 × 4.2 × 4.0 cm firm pink-tan to white lobulated mass (Fig. 4a, b). The mass had a whorled bulging cut surface and was well encapsulated. The mass extended into the wall along the posterior, superior, inferior, and medial aspect of the IVC (Fig. 4c). The tumor measured 2.0 cm from the renal sinus. Representative sections of the superior, inferior and retroperitoneal margins of the IVC and mass were submitted for frozen section diagnosis. No areas of ulceration were grossly noted. On sectioning the kidney, the cut surfaces showed unremarkable cortical medullary junctions with a cortical thickness averaging 0.6 cm. The renal capsule was intact and unremarkable. No enlarged or palpable lymph nodes were noted in the renal hilum nor surrounding the IVC.

A uterus was also submitted to pathology which was 229 grams and 6.5 × 6.0 × 5.5 cm in dimensions. On sectioning, four firm irregular intramural masses were noted ranging from 1.2 cm up to 3.2 cm in greatest dimension. The cut surface of each was homogenous pink-tan to white whorled and bulging, grossly consistent with leiomyomata. No areas of calcification, hemorrhage, necrosis, or cystic degeneration were noted.

**Figure 5:**
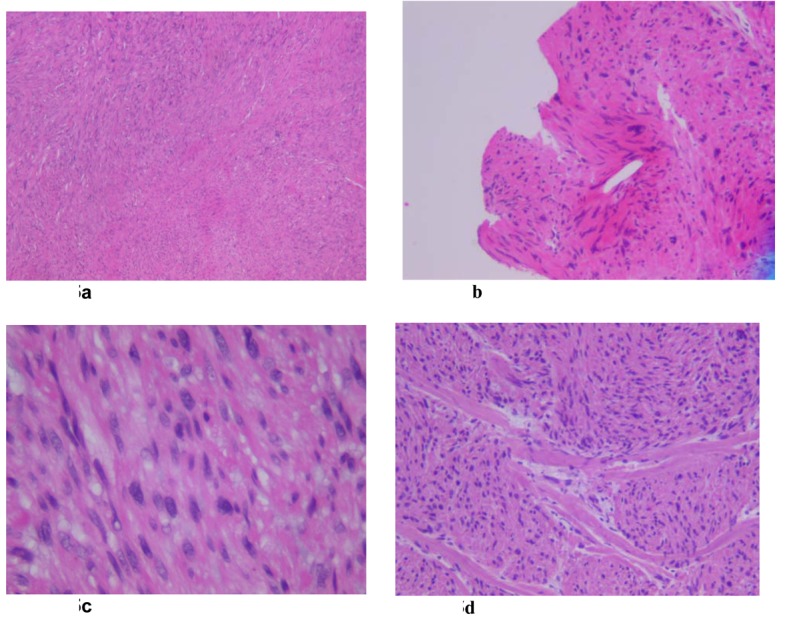
(a - d): The malignant spindle cells have elongated eosinophilic cytoplasm and elongated nuclei with rounded ends. The cells are arranged in fascicles. There is notable focal increased cellularity with mild to moderate nuclear atypia. The mitotic rate is 7/10 high power fields, with focal microscopic necrosis.

Microscopic evaluation of the tumor revealed malignant spindle cells with marked nuclear atypia. The malignant spindle cells had elongated eosinophilic cytoplasm and elongated nuclei with rounded ends. The mitotic rate was 7/10 high power fields, with focal microscopic necrosis. The cells were arranged in fascicles (Fig. 5a-d).

The patient had an unremarkable postoperative course and was discharged the following week. A limited non diagnostic post surgical CT scan was performed for placement of radiation therapy. No radiological evidence of lung metastases or lymphadenopathy was noted.

Radiation therapy began with 10 mV photons being prescribed, 5400 cGy was delivered in 30 equal fractions and inverse treatment planning techniques were utilized to maximize dose to the target and minimize dose to the normal surrounding tissues, particularly the left kidney. Daily image-guided radiation therapy was utilized to facilitate precision of beam delivery.

The patient tolerated adjuvant radiation therapy well. She is presently without clinical evidence of disease and is scheduled for repeat CT of the chest, abdomen and pelvis for restaging purposes and treatment course.

## Discussion

Leiomyosarcomas are the second most common primary retroperitoneal tumor in adults [1, 8], 70-90% of the primary retroperitoneal tumors in adults are malignant. Leiomyosarcoma of the IVC are rare soft tissue sarcomas which account for only 0.5% of all soft tissue sarcomas in adults. However, it is the most common primary malignancy of the inferior vena cava. Fewer than 300 cases are reported in literature till date [1].

### Pathologic features

Pathological features of the IVC leiomyosarcoma help in understanding clinical and radiological features better. In majority of retroperitoneal leiomyosarcomas growth is extrinsic to blood vessels. Tumors expand along tissue planes of least resistance often displacing adjacent organs. Direct invasion of the viscera is rare at least initially. The tumor is circumscribed by a pseudocapsule formed when the expanding tumor compresses adjacent organs. Intraluminal leiomyosarcomas are smaller than those that are extravascular. Often they present as nodular or polypoid masses attached to the vessel wall firmly. The cut section has a whitish gray whorled appearance. The intraluminal growth expands the IVC diameter, at times extending into the right heart and even into the pulmonary artery. Leiomyosarcomas with extraluminal and intraluminal growth pattern show great variation in the size of individual components. The finding of intra and extraluminal growth pattern is unique to leiomyosarcoma compared to other primary retroperitoneal tumors [3].

Leiomyosarcoma on a microscopic level present a typical pattern of interlacing bundles of spindle shaped cells with blunt ended nuclei. Metastases to the liver, lungs and lymph nodes usually occur late. Recurrences are common, often exhibiting more aggressive histologic features than the original neoplasm [3].

Leiomyosarcoma of the IVC are often limited in clinical expression. The main presenting symptoms are abdominal pain, a palpable mass and lower extremity edema. Symptoms vary according to dimension of the tumor, growth pattern and localization of the tumor [9, 10].

Suprahepatic leiomyosarcoma generally present with intraluminal venous extension and may result in Budd Chiari syndrome with hepatomegaly, jaundice and massive ascites [7]. The right heart and pulmonary artery may be involved. Nausea, vomiting and lower extremity edema may be present. Middle segment tumors may present with right hypochondrium and epigastric pain simulating hepatobiliary disease. Extension to suprahepatic veins may present with symptoms of Budd Chiari syndrome, while renal dysfunction results from extension into renal vessels [11-13]. Tumors of the lower segment of the IVC, which usually grow extraluminally can present with hypogastric and right iliac fossa pain [11, 13]. Lower extremity edema is common.

Noninvasive imaging modalities such as CT scan, USG and MRI are often used to diagnose these tumors. They provide valuable information regarding their origin, evaluating the presence of local invasion and excluding distant metastases. The sensitivity and specificity of CT in assessment of the tumors are 78% and 96% respectively and even higher with MRI (95-100%) [14]. Ascending cavography can delineate the involvement of renal and hepatic veins and allow biopsy of the tumor. Selective arteriography of the celiac trunk may also be performed in patients where hepatic invasion or metastasis is suspected and transesophageal echocardiography can exclude or verify intracardial tumor extension [14]. Percutaneous venous biopsy with CT or echo guidance provides pathological confirmation.

### Operative management

Multivisceral resection including the IVC is a technically demanding procedure but can be useful to allow extension of a potentially curable resection. Clinical conditions requiring resection of the inferior vena cava (IVC) are rare. Traumatic or iatrogenic injury, chronic post-thrombotic or membranous occlusion, and malignancy are the main indications. The notable malignant conditions include renal cell carcinoma, Wilms tumor, leiomyosarcoma, adrenal tumor, hepatic carcinoma, and retroperitoneal metastatic lymph nodes from testicular carcinoma [7].

Operative management for tumors involving segment II and especially segment III are challenging. If the superior border of the tumor does not extend beyond the inferior hepatic edge, an abdominal approach is usually used, either by conventional laparotomy or preferably by right subcostal incision [4].

If the subcostal approach is used, exposure of the infrahepatic and retrohepatic IVC can be optimized by extending the incision into a lombotomy with the patient in a slightly lateral decubitus position [4]. A thoracoabdominal approach is preferred if the tumor extends to the retrohepatic or suprahepatic part of the IVC.

Kieffer et al mentions of the advantage of combining laparotomy with sternotomy over thoracophrenotomy [4]. With midline incision of the phrenic center, this route gives good exposure of the suprahepatic IVC as well as of the retrohepatic IVC after sectioning of the hepatic ligaments. Sternotomy combined with laparotomy has the added benefit of providing good conditions for establishing cardiopulmonary bypass for treatment of intracardiac extension if present [4, 6].

Some authors recommend wide tumor resection at a safe distance from the tumor [4, 8]. Partial resection of the IVC followed by direct suture or prosthetic patch angioplasty is rarely curable. Complete resection of the IVC is mandated in most cases.

Simple ligation is possible after complete or subtotal resection of the infra-renal IVC (segment I) and/or retrohepatic portion of the IVC (segment II) in association with resection of the right kidney. There is a difference in collateral circulation between the left and the right. Collaterals on the left (capsular, genital, reno-azygo-lumbar veins) are generally sufficient for satisfactory venous return without occurrence of renal insufficiency. On the contrary, on the right there are no effective collaterals [4].

In cases of incomplete obstruction of the IVC, ligation may be required especially if the procedure involves the gastrointestinal tract, whereby the risk of prosthetic infection is high. Pressure monitoring is performed in such cases to rule out excessive venous hypertension during clamping. As a general rule, a proximal pressure reading of 30 mm Hg or more indicates caval reconstruction to avoid postoperative edema of the lower extremity.

Ringed reinforced ePTFE graft is prosthesis of choice for IVC replacement. It provides the best results, given the length of the missing segment and the need for strength to resist compression in the abdomen. Collapse of the graft may be an important factor in thrombosis. Many authors prefer a 20-mm-diameter graft for best congruency with the native vessel [7, 8, 10]. Others recommend smaller grafts (14 - 16 mm) for infrarenal replacement to increase blood velocity [10, 15].

Some authors strongly recommended the creation of an arteriovenous fistula to ensure patency. The fistula eliminates the need for long-term anticoagulation therapy.

For infrahepatic IVC reconstruction simple clamping is often adequate. Complications such as arterial hypotension or proximal venous hypertension are rare and can be dealt with by associated clamping of the infrarenal aorta or supraceliac aorta. Vascular exclusion, indicated for tumors extending to the retrohepatic portion of the IVC allows to perform the upper anastomosis of the prosthesis near the infrahepatic veins so that hepatic circulation can be reestablished quickly thus reducing hepatic ischemia time [4, 11]. Vascular exclusion also makes it possible to perform associated right or left extended hepatectomy.

For surgical treatment of tumors involving the suprarenal segment of the IVC venovenous shunting with selective hypothermic hepatic perfusion may be helpful [4, 12, 13]. These modalities provide adequate time cushion for anastomosis of the prosthesis flush with the right atrium and for reimplantation of a patch containing the suprahepatic veins.

Reports of long-term patency have been well documented in literature without anticoagulation although in our case we decided to administer anticoagulation therapy [10, 15]. Infection of synthetic grafts is a concern. Omental interposition between the graft and resected viscera may be beneficial.

In rare cases involving tumors with intracardiac extension, cardiopulmonary bypass may be required. Venous cannulations should be placed through the tip of the right atrium for the territory drained by the SVC and in the femoral vein or infrarenal IVC for the territory drained by the IVC. Some authors prefer to perform hypothermic circulatory arrest at 18 °C rather than normothermic cardiopulmonary bypass because the former provides a blood-free operating field.

Right nephrectomy is often required for tumors involving segment II of the IVC, even if the kidney is not directly involved. If the tumor only brushes the ostium of the right kidney vein, autotransplantation of the right kidney can be performed into the right iliac fossa. This procedure requires prosthetic reconstruction of the IVC.

Aggressive management of advanced abdominal tumors can produce long-term survival. The 5-year actuarial survival rate after curative resection exceeds 50% for renal cell carcinoma with caval extension and 28% for primary leiomyosarcoma [7, 10, 15-17]. In the present series, estimated median survival was 37 months for patients with malignant disease. On the other hand, median survival without resection is 1 month for patients with primary leiomyosarcoma [7, 15].

### Adjuvant Therapy/XRT

The use of adjuvant treatment has evolved over the last decade. Contrary to prior belief, at the present time, radical resection followed by adjuvant chemotherapy is considered the optimal therapeutic strategy for tumors without metastasis at the time of initial diagnosis. Hines et al suggests that new radiation therapy techniques, especially applicable to tumors involving the infrahepatic part of the IVC, may prolong survival after surgical resection [5].

Neoadjuvant therapy may be given to downsize the tumor and increase resectability rates. Nonetheless, when complete resection is not possible, debulking combined with radiation therapy still provides good palliation [2, 18].

In another series, 14 patients with leiomyosarcoma of the IVC were treated with wide resection between 1978 and 1997; in this series, radiation therapy diminished local recurrence and improved median survival (6 months in 2 patients without irradiation compared to 51 months in 12 irradiated patients) [5, 18]. Patients who received combined chemotherapy and radiation lived longer than those who received radiation therapy alone [5, 18].

Chemotherapy, using adriamycin- ifosfamide or chemotherapy combined with

radiation therapy offer potential adjuvant treatment options [5, 18]. The 5-year cumulative survival rate was 53% for patients with leiomyosarcoma of the IVC suggesting that aggressive surgical management combined with adjuvant therapy offers the best treatment for patients with leiomyosarcoma of the IVC [5, 18].

### Prognosis

Even with radical surgery, 5- and 10-year survival of 49.4% and 29.5% respectively has been reported [19]. With recurrence rates as high as 50% reported in some literature, surgery may simply be providing palliation in many cases [19]. Tumors of the retrohepatic IVC tend to present earlier as a result of pressure exerted on surrounding structures. A superior prognosis with 5- and 10-year survival rates of 56.7% and 47.3% are reported [19]. Abdominal pain, the clinical presence of a mass and the ability to achieve clear resection margins were noted to be favorable factors identified by the registry whereas poor prognostic indicators include high-grade tumors, suprahepatic tumors, and presentation with IVC occlusion or Budd-Chiari syndrome.
